# Coping Strategies and Social Support for Transition Readiness Among Youth With Sickle Cell Disease

**DOI:** 10.1001/jamanetworkopen.2026.22753

**Published:** 2026-07-13

**Authors:** Apoorva S. Iyengar, Tanisha Belton, Jack Chen, Tisheya Ward, Olivia Teng, Banu Aygun, Abena Appiah-Kubi, Nataly Apollonsky, Donna Boruchov, Biree Andemariam, Omar Niss, Lori E. Crosby, Lisa Schwartz, Lamia Barakat, Kim Smith-Whitley, Sophia Jan, Caren Steinway

**Affiliations:** 1 Department of Pediatrics, Northwell Health, New Hyde Park, New York; 2 Department of Pediatrics, Cohen Children’s Medical Center, New Hyde Park, New York; 3 PolicyLab, Division of General Pediatrics, Children’s Hospital of Philadelphia, Pennsylvania; 4 Drexel University College of Medicine, Philadelphia, Pennsylvania; 5 St Christopher’s Hospital for Children, Philadelphia, Pennsylvania; 6 Connecticut Children’s Medical Center, Hartford; 7 New England Sickle Cell Institute, University of Connecticut Health, Farmington; 8 Department of Pediatrics, Cincinnati Children’s Hospital Medical Center, Ohio; 9 Division of Hematology, Cincinnati Children’s Hospital Medical Center, Ohio; 10 Behavioral Medicine and Clinical Psychology, Cincinnati Children’s Hospital Medical Center, Ohio; 11 Division of Oncology, Children’s Hospital of Philadelphia, Pennsylvania; 12 Division of General Pediatrics, Children’s Hospital of Philadelphia, Pennsylvania

## Abstract

**Importance:**

Suboptimal transitions from pediatric to adult health care can negatively impact disease outcomes, medication regimen adherence, and disease self-management in adulthood. Little is known about whether the presence of coping strategies in young adults (YAs) with sickle cell disease (SCD) is associated with higher transition readiness scores.

**Objective:**

To evaluate whether coping strategies and social support are associated with higher transition readiness scores in YAs with SCD.

**Design, Setting, and Participants:**

This cross-sectional study was a subanalysis of baseline data from a prospective randomized clinical trial of YAs aged 17 to 25 years with SCD planning to transition to an adult sickle cell clinic within the next 12 months at 5 institutions across Connecticut, New York, Ohio, and Pennsylvania. The recruitment period was from January 15, 2019, to December 31, 2022. Analysis used data from this time period and was conducted from September 30, 2024, to June 30, 2025.

**Main Outcomes and Measures:**

Measures included the Transition Readiness Assessment Questionnaire (TRAQ) to assess transition readiness, the Medical Outcomes Study–Social Support Survey (MOS-SSS) emotional/informational subscale to assess social support, and the Brief-COPE (problem-focused, emotion-focused, and avoidant subscales) to assess coping strategies. Bivariate and multivariable linear regression analyses were used to assess differences of transition readiness. Covariates included worry (PedsQL Sickle Cell Disease Module worry I), age, gender, SCD disease severity, and social support.

**Results:**

The final cohort included 373 YAs (mean [SD] age, 18.9 [1.9] years; 190 female [51.5%]), with 335 of 367 reporting race as Black (91.3%) and 335 of 365 reporting ethnicity as non-Hispanic/Latino (91.8%). Unadjusted analysis found that overall coping (mean difference, 0.14; 95% CI, 0.01-0.27; *P* = .03), problem-focused coping (mean difference, 0.18; 95% CI, 0.09-0.27; *P* < .001), and the emotional/informational subscale of the MOS-SSS (mean difference, 0.13; 95% CI, 0.07-0.19; *P* < .001) were associated with higher transition readiness scores. Adjusted linear regression analysis showed that problem-focused coping (mean difference, 0.10; 95% CI, 0.003-0.19; *P* = .04) was associated with transition readiness even after adjusting for age, gender, disease severity, worry, recruitment site, and social support.

**Conclusions and Relevance:**

In this cross-sectional study in a national cohort of adolescents and YAs with SCD, problem-focused coping strategies were associated with higher levels of transition readiness. These findings suggest important intervention targets for supporting transitioning adolescents with SCD.

## Key Points

**Question:**

How are coping strategies and social support associated with self-reported transition readiness in young adults with sickle cell disease (SCD) transitioning to adult care?

**Findings:**

In this cross-sectional study using baseline data from a randomized trial of 373 young adults with SCD (aged 17-25 years), problem-focused coping and social support were associated with higher transition readiness from pediatric to adult hematology care.

**Meaning:**

These findings suggest that young adults with SCD with higher levels of problem-focused coping skills and social support are more likely to have higher transition readiness scores.

## Teaser

This cross-sectional study explores whether coping strategies and social support are associated with readiness to transition to adult health care in young adults with sickle cell disease.

## Introduction

Sickle cell disease (SCD) is an autosomal recessive blood disorder with multisystemic effects affecting approximately 100 000 people in the US and primarily affecting patients of African descent.^1^ The mutation in SCD causes an abnormal production of sickle hemoglobin, which assumes a crescent or sickle-like shape.^2^ Sickled cells can lead to vaso-occlusion with several associated complications, including pain crises, acute chest syndrome, splenic sequestration, and stroke, requiring close medical monitoring and prompt pain management. Improvements in SCD diagnosis and treatment have led to higher life expectancy in the past century.^3^ Children with SCD have improved survival rates, with more than 95% of patients surviving into adulthood in the US.^4^ With more children surviving into adulthood, the focus on improving the transition between pediatric and adult care becomes increasingly important.

The transition from pediatric to adult care is the purposeful planned movement of adolescents and young adults (YAs) from child-centered to adult-oriented health care systems.^5^ Suboptimal transitions have been associated with poorer disease outcomes, higher health care utilization, poorer medication regimen adherence, and poor overall success in adulthood.^6^ However, many YAs who have transitioned or are actively transitioning to adult care note various barriers, including lack of timely care in adult settings, negative perceptions about adult care, as well as several stigmas that are associated with SCD impairing health care interactions and promoting overall negative physiological and psychosocial well-being.^7^ Given that most patients with SCD are of African descent and approximately 60% of patients are receiving Medicaid,^8^ there are also unique socioeconomic barriers and structural inequities,^9^ including racial bias, insurance status, and financial instability, which can further impede appropriate care and transitions of care that these YAs deserve. Notably, prior studies identified that YAs may disengage with care during the transition period for multifaceted reasons. Prussien et al,^10^ however, commented that acceptance of an SCD diagnosis can be a facilitator of patient engagement. Through that study it was found that patients, caregivers, and practitioners alike all noted that self-efficacy, patient skills, and strong relationships with clear communication were mechanisms that could help patients navigate an often inequitable health care system. Other studies have also emphasized the importance of self-advocacy as an essential skill in adult-care spaces.^11^ Further studies noted the need to encourage learning self-advocacy skills to assume responsibility in building trusting therapeutic relationships and complete health maintenance tasks.^12^

The YA time period is a time for new life experiences and increasing autonomy requiring comprehensive disease knowledge for patients and strong self-management skills.^13^ Adolescents must be supported during this period of transition to ensure that patients experience the best medical outcomes. Thus, efforts in recent years have focused on methods to improve overall transitions for YAs with SCD and other conditions.

Previous research^14^ found that positive coping strategies are associated with higher transition readiness scores in various chronic conditions. It has also been shown that short-term efficacy of use of transition navigators to provide support and disease-specific information for patients with SCD transitioning to adult care.^15^ The amount of disease-specific worry was thought to influence the relationship between coping strategies and transition readiness.^14^ Patients who are older are thought to be more adept at developing coping strategies and have better transition readiness. However, to our knowledge, specific data evaluating the relationship between coping and transition readiness in patients with SCD have not been examined. In older adults, poorer social supports, such as single-parent households, were associated with higher levels of social vulnerability and overall morbidity.^16^ To our knowledge, emotional or educational social support on transition readiness in patients with SCD also has not been examined.

The primary aim of this study was to evaluate whether the presence of coping strategies in YAs with SCD is associated with higher transition readiness scores in a large national sample baseline dataset and which coping strategies had the greatest impacts. We hypothesized that patients with positive coping strategies and better social support will have higher transition readiness scores and, in turn, be more prepared to adjust to adult care.

## Methods

### Study Design and Patient Population

This cross-sectional study used baseline data from the Community Health Workers and Mobile Health for Improving Transition to Adult Sickle Cell Disease Care Trial (COMETS study), a randomized clinical trial comparing the following interventions of using community health workers, mobile health applications, and enhanced usual care to understand how to ease the transition period between pediatric and adult care for YAs with SCD.^17^ The study was funded in September 2017. Institutional review board approval was received in June 2018 at the primary institution, The Children’s Hospital of Philadelphia (CHOP), and submitted to each participating site for site-level approach. For the present study, the Northwell Health Institutional Review Board waived the need for review and informed consent as a secondary analysis of data from a previous trial.

Participants aged 17 to 28 years received SCD care followed at 1 of the 5 institutions: CHOP, St Christopher’s Hospital for Children (St Chris), Cincinnati Children’s Hospital and Medical Center (CCHMC), Cohen Children’s Medical Center (CCMC), and Connecticut Children’s Medical Center (CT Children’s). All participants planned to transition to an adult SCD clinic within 12 months. Informed consent was obtained from all study participants prior to their involvement in the research by the study coordinators. Comprehensive information about the study’s objectives, procedures, potential risks, and the participants’ rights, including the option to withdraw at any time without consequence, were provided to participants. The outcomes of health care–related quality of life (HRQOL) and secondary outcomes of disease self-management and engagement in the adult health care system were analyzed. This study presents a secondary analysis of completed surveys at the baseline time point between January 2019 and December 2022, among all participants in the COMETS trial. Analysis was conducted from September 30, 2024, to June 30, 2025. The Strengthening the Reporting of Observational Studies in Epidemiology (STROBE) reporting guideline was followed.

### Measures

Measures utilized include the Transition Readiness Assessment Questionnaire (TRAQ),^18,19^ Medical Outcome Study–Social Support Survey (MOS-SSS) emotional/information subscale,^20^ Brief Coping Orientation to Problems Experienced or Brief-COPE^21,22^ (problem-focused, emotion focused, and avoidant subscales), and the PedsQL SCD module.^23,24^ In addition, items on demographic characteristics (age, race, and ethnicity) were collected by self-report through the baseline surveys. Race and ethnicity information was collected to capture overall characteristics of the data.

### TRAQ

The TRAQ^18,19^ is a non–disease-specific validated questionnaire with 20 items rated on a 5-point Likert scale that were used to assess transition readiness.^19^ Each item pertains to 1 of 5 domains (managing medications, appointment keeping, tracking health issues, talking with clinicians, and managing daily activities). Responses to each item consisted of (1) “No, I do not know how,” (2) “No, but I want to learn,” (3) “No, but I am learning to do this,” (4) “Yes, I have started to do this,” and (5) “Yes, I always do this when I need to.” Total and subscale scores were averaged among the items, with higher scores indicating greater transition readiness. Validity studies have found higher self-advocacy TRAQ scores for females and older YAs as well as higher self-management TRAQ domain scores for older YAs.^19,25^

### MOS-SSS

The MOS-SSS^20^ is a 19-item survey, rated on a 5-point Likert scale, used to understand the perceived availability of social support on 4 subscales: emotional/informational, tangible, affectionate, and positive social interaction. Our study focused on the 8-item emotional/informational subscale to understand social support and to explore modifiable interventions for this transition period. Responses consisted of (1) “None of the time,” (2) “A little of the time,” (3) “Some of the time,” (4) “Most of the time,” and (5) “All of the time.” The total score was averaged among the items, with higher scores indicating higher levels of self-reported social support in YAs. This subscale focuses on positive expressions of positive effects and the use of advice or information.

### Brief-COPE

The Brief-COPE^21,22^ is a 28-item measure used to evaluate coping strategies among the participants. The total and subscale (problem-focused, emotion-focused, and avoidant subscales) composite scores were based on an average of the 4-point Likert scale. Responses consisted of (1) “I haven’t been doing this at all,” (2) “I’ve been doing this a little bit,” (3) “ I’ve been doing this a medium amount,” and (4) “I’ve been doing this a lot.” The 8-item problem-focused coping subscale is characterized by using active coping strategies that require the synthesis of information and positive reframing. Participants in prior studies with higher scores in this category have been noted to have more psychological resilience and problem-solving capabilities.^26^ The 10-item emotional coping subscale has been described as using venting, emotional support, or self-blame. The 8-item avoidant coping subscale is characterized by denial, substance use, and behavioral disengagement, which have been shown to be the most maladaptive behaviors.

### PedsQL SCD Module

The PedsQL SCD module^23,24^ is a validated 43-item tool that assesses pain, emotional health, treatment experience, and communication on 9 scales: pain and hurt (9 items), pain impact (10 items), pain management and control (2 items), worry I (5 items), worry II (2 items), emotions (2 items), treatment (7 items), communication I (3 items), and communication II (3 items). Scores are based on a 5-point Likert response scale (0 = never a problem, 1 = almost never a problem, 2 = sometimes a problem, 3 = often a problem, 4 = almost always a problem). Responses are then reverse-scored to a 0 to 100 scale (0 = 100, 1 = 75, 2 = 50, 3 = 25, 4 = 0). The final scale score is the average of the items, with higher scale scores indicating better HRQOL and fewer SCD symptoms and problems. For this study we looked at disease-specific worry (worry I, worry II, and emotions subscale), which are important aspects to mental health and quality of life.

### Covariates

Covariates included worry (PedsQL SCD module worry I),^23^ age, sex, disease severity, recruitment site, and social support regarding the relationship between coping strategies and transition readiness. Quality of life in prior studies has been shown to have a relationship with improved transition readiness.^27^ Older age has previously been associated with higher levels of transition readiness.^25,28^ Patients who are in the midst of the transition have also highlighted the need for social support in transition.^29^ While disease-specific worry has not been suggested to be associated with transition readiness in prior studies, we hypothesized that there would be an inverse relationship with transition readiness.

Disease severity was categorized as moderate or severe based on the idea that SCD can never be thought of as less moderate. Severe SCD was defined as any history of acute chest syndrome, stroke, 3 or more emergency department visits in the last 3 years, prescribed hydroxyurea, or receipt of monthly exchange transfusion therapy.^23,30,31^ We hypothesized that YAs with higher SCD severity may have more barriers to better coping strategies and transition readiness.

### Statistical Analysis

Descriptive statistics (frequencies and percentages), along with means and SDs of overall coping scores, were used to profile the cohort by demographic characteristics. Unadjusted linear regressions examined the associations between the Brief-COPE, its subscales, worry, and social support with transition readiness. An adjusted multivariable linear regression was conducted to identify associations between problem-focused coping and transition readiness with all the covariates mentioned above. All analyses were performed using IBM SPSS version 27 (IBM Corporation) and 2-sided *P* ≤ .05 was considered statistically significant.

## Results

The final cohort comprised 373 YAs. The cohort had a mean (SD) age of 18.9 (1.9) years, an almost equal distribution of males and females (females, 190 [51.5%]; males, 179 [48.5%]), with 335 of 367 reporting race as Black (91.3%) and 335 of 365 reporting ethnicity as non-Hispanic or Latino (91.8%). Other race and ethnicity categories were American Indian and Alaska Native, 0; Asian, 0; multiracial, 15 (4.1%); Hispanic or Latino, 30 (8.2%); Native Hawaiian and Pacific Islander, 1 (0.3%); and other (no further breakdown available), 14 (3.8%). More than half of the sample had severe disease. There were no significant differences in coping scores among demographic characteristics (Table 1).

**Table 1.** Characteristics of the Study Population

Characteristic	Total cohort (N = 373)	Mean (SD)	
		Brief-COPE scores (n = 372)	Transition readiness scores (n = 372)
Demographic characteristics			
No.	370	369	369
Age, y			
Mean (SD)	18.9 (1.9)	NA	NA
≤21, No. (%)	336 (90.8)	2.3 (0.5)	3.7 (0.7)
>22, No. (%)	34 (9.2)	2.4 (0.6)	4.1 (0.5)
Sex, No. (%)	369		
Male	179 (48.5)	2.3 (0.6)	3.7 (0.7)
Female	190 (51.5)	2.4 (0.5)	3.8 (0.7)
Race,^a^ No. (%)	367		
American Indian and Alaska Native	0	0	0
Asian	0	0	0
Black	335 (91.3)	2.4 (0.5)	3.7 (0.7)
Multiracial	15 (4.1)	2.3 (0.6)	3.9 (0.7)
Native Hawaiian and Pacific Islander	1 (0.3)	NA	NA
White	2 (0.5)	2.4 (0.6)	4.2 (0.6)
Other^b^	14 (3.8)	2.0 (0.6)	4.1 (0.6)
Ethnicity,^a^ No. (%)	365		
Hispanic/Latino	30 (8.2)	2.1 (0.6)	3.9 (0.6)
Non-Hispanic or Latino	335 (91.8)	2.4 (0.5)	3.7 (0.7)
Recruitment site, No. (%)			
CHOP	143 (38.3)	2.3 (0.5)	3.8 (0.6)
CCHMC	35 (9.4)	2.5 (0.6)	3.9 (0.7)
CT Children’s	43 (11.5)	2.3 (0.5)	3.6 (0.8)
CCMC	91 (24.4)	2.3 (0.5)	3.6 (0.7)
St Chris	61 (16.4)	2.4 (0.6)	3.8 (0.6)
History of intellectual disability, No. (%)			
Yes	18 (4.8)	2.5 (0.7)	3.7 (0.8)
No	355 (95.2)	2.3 (0.5)	3.8 (0.7)
History of stroke or acute chest syndrome, No. (%)			
Yes	143 (38.3)	2.3 (0.5)	3.7 (0.7)
No	230 (61.7)	2.4 (0.5)	3.8 (0.6)
Prescribed hydroxyurea, No. (%)			
Yes	187 (50.1)	2.4 (0.6)	3.8 (0.7)
No	186 (49.9)	2.3 (0.5)	3.7 (0.6)
Disease severity, No. (%)			
Moderate	164 (44.0)	2.3 (0.5)	3.8 (0.6)
Severe^c^	209 (56.0)	2.4 (0.5)	3.7 (0.7)

Abbreviations: CCHMC, Cincinnati Children’s Hospital and Medical Center; CCMC, Cohen Children’s Medical Center; CHOP, Children’s Hospital of Philadelphia; CT Children’s, Connecticut Children’s Medical Center; NA, not applicable; St Chris, St Christopher’s Hospital for Children.

^a^ Information on race and ethnicity was collected by self-report through the baseline surveys.

^b^ Information on races classified as other was not collected.

^c^ Severe sickle cell disease was defined as any history of acute chest syndrome, stroke, ≥3 emergency department visits in the last 3 years, prescribed hydroxyurea, or receipt of monthly exchange transfusion therapy.

Overall, the sample exhibited high transition readiness (mean [SD], 3.7 [0.7]), high social support (mean [SD], 3.6 [1.1]), and problem-focused coping strategies were most frequently used (mean [SD], 2.7 [0.7]). Unadjusted analyses found that greater use of overall coping strategies and problem-focused coping strategies (overall coping strategies: mean difference, 0.14; 95% CI, 0.01-0.27; *P* = .03; problem-focused coping strategies: mean difference, 0.18; 95% CI, 0.09-0.27; *P* < .001), and having more social support (mean difference, 0.13; 95% CI, 0.07-0.19; *P* < .001) were associated with higher transition readiness scores (Table 2). After adjusting for other covariates, we found that greater use of problem-focused coping strategies was a significant factor in transition readiness, increasing the score by 0.10 units (95% CI, 0.003-0.19; *P* = .04). Additional factors in transition readiness after adjusting for other variables included age (for every 1-year increase, transition readiness is expected to increase by 0.12 units; 95% CI, 0.09-0.16; *P* < .001), sex (being female was expected to increase transition readiness by 0.17 units; 95% CI, 0.04-0.30; *P* = .01), and social support (greater social support was expected to increase transition readiness by 0.11 units; 95% CI, 0.05-0.17; *P* < .001) (Table 2).

**Table 2.** Unadjusted and Adjusted Analyses of Associations Between Coping Strategies and Transition Readiness

Variable	Unadjusted analysis		Adjusted analysis	
	Mean difference (95% CI)	*P* value	Mean difference (95% CI)	*P* value
QOL (worry)	0.001 (–0.002 to 0.004)	.65	0.00 (–0.001 to 0.001)	.79
Brief-COPE overall	0.14 (0.01 to 0.27)	.03	NA	
Problem-focused	0.18 (0.09 to 0.27)	<.001	0.10 (0.003 to 0.19)	.04
Emotion-focused	0.04 (–0.07 to 0.16)	.44	NA	
Avoidant	0.03 (–0.09 to 0.16)	.60	NA	
MOS-SSS scale^a^	0.13 (0.07 to 0.19)	<.001	0.11 (0.05 to 0.17)	<.001
Age	NA	NA	0.12 (0.09 to 0.16)	<.001
Sex^b^	NA	NA	0.17 (0.04 to 0.30)	.01
Disease severity^c^	NA	NA	–0.05 (–0.18 to 0.08)	.46
Site^d^				
CCHMC	NA	NA	–0.01 (–0.24 to 0.22)	.92
CT Children’s	NA	NA	–0.19 (–0.40 to 0.03)	.08
CCMC	NA	NA	–0.08 (–0.25 to 0.09)	.37
St Chris	NA	NA	–0.06 (–0.25 to 0.13)	.56
Model summary	NA	NA	NA	<.001

Abbreviations: CCHMC, Cincinnati Children’s Hospital and Medical Center; CCMC, Cohen Children’s Medical Center; CHOP, Children’s Hospital of Philadelphia; CT Children’s, Connecticut Children’s Medical Center; MOS-SSS, Medical Outcomes Study Social Support Survey; NA, not applicable; QOL, quality of life; St Chris, St Christopher’s Hospital for Children.

^a^ Emotional/informational subscale.

^b^ Male vs female.

^c^ Moderate vs severe.

^d^ Reference = CHOP.

## Discussion

Our study had 2 findings with notable implications for clinical practice. First, in unadjusted analyses, overall coping, problem-focused coping, and emotional/informational support were all independently associated with better transition readiness. YAs who reported higher levels of overall and problem-focused coping showed greater preparedness for transition, as measured by TRAQ scores, while the finding on social support, as measured by the MOS-SSS subscale, extends beyond previous literature and highlights the crucial role of both practical knowledge and emotional supports in preparing YAs for transition.

Interestingly, despite overall coping being associated with transition readiness, neither emotion-focused nor avoidant-focused coping was associated with transition readiness, suggesting the fundamental importance of problem-focused coping strategies in transition preparation. Therefore, only problem-focused coping was included in the adjusted analyses.

Second, the association between problem-focused coping and transition readiness was found even after adjusting for disease severity, worry, age, gender, and social support. This finding is particularly noteworthy because it suggests that problem-focused coping strategies may be more influential than disease-specific factors in determining transition readiness. Further, demographic factors also play a significant role, with older age and female gender being associated with higher transition readiness scores. The age finding aligns with previous research and developmental expectations.^25,28^ Although the gender difference has been noted in prior TRAQ validity studies, it differs from studies showing no gender differences.^19,25,28,32^

These findings align with and extend current literature. A previous study evaluating children aged 13 ± 2 years with various chronic medical conditions found that positive coping strategies were related to lower anxiety scores and higher levels of transition readiness.^14^ However, our study is the first, to our knowledge, to show this association specifically in a large cohort of YAs with SCD, while also identifying the distinct importance of problem-focused coping strategies. Previous research has evaluated how disease-related risk factors and psychosocial factors contribute to health care transition readiness. One study^33^ of 41 YAs with chronic kidney disease aged 13 to 18 years found that family cohesion was associated with transition readiness, in parallel to our finding regarding social support. This relationship has previously been described by the Social-ecological Model of Adolescent and Young Adult Readiness to Transition (SMART) model.^34^ This model described a relationship between 6 modifiable facets, including relationships/communication and skills/self-efficacy as key factors, in successful transitions for pediatric oncology patients. Our study’s larger sample size and focus on SCD provides stronger evidence for the role of both social support and coping strategies in transition readiness and aligns with the SMART model.

Our findings suggest several critical intervention targets for improving transition outcomes. First, health care teams should implement routing screening for coping mechanisms and social support during adolescent clinic visits. Second, health care teams should develop structured programs to teach problem-focused coping skills. Third, health care teams should enhance social support through peer mentoring and transition navigation programs. Finally, health care teams should provide targeted support for younger patients and those who may have less effective coping strategies. Our findings on the importance of social support and problem-focused coping align with and inform ongoing intervention research, particularly the COMETS study.^35^ COMETS tests the impact of mobile health applications and community health workers to aid YAs with SCD in the transition from pediatric to adult care, directly assessing the gaps in social support and coping skill development identified in our study.^35^ Understanding how such interventions interact with individual coping styles and existing support systems could help refine and personalize transition support programs.

### Limitations

This study has limitations, mostly from the unknown directionality. Although our study allows us to highlight the associations between variables, no causality can be inferred. Future longitudinal research should examine whether interventions to improve coping skills lead to better transition outcomes. Another limitation was that all data were collected before or during the height of the COVID-19 pandemic. This timing may have influenced both coping behaviors and social support systems, potentially highlighting the importance of formal support structures during periods of social disruption. It should also be highlighted that the majority of the patients who failed transition to an adult clinic are often lost to follow-up prior to 15 years of age. The scope of the overarching study was limited to patients at the immediate transition age of 17 to 28 years, but perhaps further studies should include young adolescents to ensure higher retention to adult care. Additionally, the use of the TRAQ measure does not necessarily relate to transition outcome data at the baseline time point. Further study may be beneficial to explore this relationship.

## Conclusions

This cross-sectional analysis of a large national cohort suggests associations between problem-focused coping strategies and social support as key modifiable factors in transition readiness for YAs with SCD. These findings identify specific, actionable interventions for clinical practice. These include systematic screening for coping strategies and social support networks during routine care, implementing targeted problem-solving skills training, developing peer support systems and transition navigator roles, and integrating psychosocial support assessments into standard transition planning. By addressing these modifiable factors, health care teams can work to promote more equitable and favorable transition outcomes for YAs with SCD. Future research should focus on implementing and evaluating these targeted interventions, particularly in resource-limited settings (lack of adult care infrastructure), and examining their impact on long-term health outcomes. Potential longitudinal analyses could be the evaluation of how coping strategies relate to transition readiness and other outcomes over time.

## Notes

Supplement 1.
Data Sharing Statement
