# Supplementary material for: Coping Strategies and Social Support for Transition Readiness Among Youth With Sickle Cell Disease
Source: JAMA Netw Open. 2026 Jul 13;9(7):e2622753. doi: 10.1001/jamanetworkopen.2026.22753 (PMC13366194; doi:10.1001/jamanetworkopen.2026.22753)
Supplement: Supplement 1. — Data Sharing Statement [file jamanetwopen-e2622753-s001.pdf]

Data Sharing Statement

Iyengar. Coping Strategies and Social Support for Transition Readiness Among Youth With Sickle Cell Disease. *JAMA Netw Open*. Published July 13, 2026.
doi:10.1001/jamanetworkopen.2026.22753

Data
Data available: Yes
Data types: Deidentified participant data
How to access data: Deidentified individual participant data will be made available upon request. Proposals for access should be sent to sjan1@northwell.edu. The data will be shared for the purpose of further research with a signed data use agreement
When available: With publication

Supporting Documents
Document types: None

Additional Information
Who can access the data: Deidentified individual participant data will be made available upon request. Proposals for access should be sent to sjan1@northwell.edu. The data will be shared for the purpose of further research with a signed data use agreement
Types of analyses: Deidentified individual participant data will be made available upon request. Proposals for access should be sent to sjan1@northwell.edu. The data will be shared for the purpose of further research with a signed data use agreement
Mechanisms of data availability: Deidentified individual participant data will be made available upon request. Proposals for access should be sent to sjan1@northwell.edu. The data will be shared for the purpose of further research with a signed data use agreement
